# Synthesis and Characterization of Se^4+^@TiO_2_/PET Composite Photocatalysts with Enhanced Photocatalytic Activity by Simulated Solar Irradiation and Antibacterial Properties

**DOI:** 10.3390/molecules30061306

**Published:** 2025-03-14

**Authors:** Yu Ren, Rui Luan, Ziyao Zhao, Lina Tang, Chunxia Wang, Yuehui Li, Meixian Li

**Affiliations:** 1 School of Textile and Clothing, Nantong University, Nantong 226019, China; ren.y@ntu.edu.cn (Y.R.); 2315310016@stmail.ntu.edu.cn (R.L.); chloe.zhao@autoliv.com (Z.Z.); 2115110127@stmail.ntu.edu.cn (L.T.); 2 College of Textile and Clothing, Yancheng Institute of Technology, Yancheng 224051, China; cxwang@mail.dhu.edu.cn; 3 School of Microelectronics and Integrated Circuits, Nantong University, Nantong 226019, China

**Keywords:** TiO_2_, ion doping, polyester, photocatalysis properties, antibacterial properties

## Abstract

To fabricate recyclable catalytic materials with high catalytic activity, Se^4+^@TiO_2_ photocatalytic materials were synthesized by the sol–gel method. By introducing free radicals on the surface of polyester (PET) fabrics through plasma technology, Se^4+^@TiO_2_/PET composite photocatalytic materials with high photocatalytic activity were prepared. The surface morphology, crystal structure, chemical composition, and photocatalytic performance were characterized by scanning electron microscopy (SEM), X-ray diffraction (XRD), X-ray photoelectron spectroscopy (XPS), ultraviolet–visible absorption spectroscopy (UV–Vis), and photoluminescence spectroscopy (PL), respectively. The photocatalytic degradation performance was determined by assessing the degradation of azo dye methyl orange under simulated solar irradiation. The results demonstrated that Se^4+^@TiO_2_/PET exhibited a superior degradation rate of methyl orange, reaching up to 81% under simulated sunlight. The PL spectra indicated that the electron–hole pair separation rate of Se^4+^@TiO_2_/PET was higher than that of TiO_2_/PET. Furthermore, UV–Vis spectroscopy demonstrated that the relative forbidden band gap of Se^4+^@TiO_2_/PET was determined to be 2.9 eV. The band gap of Se^4+^@TiO_2_/PET was narrower, and the absorption threshold shifted toward the visible region, indicating a possible increase in its catalytic activity in simulated solar irradiation. In addition, the antibacterial properties of Se^4+^@TiO_2_/PET were subsequently investigated, achieving 99.99% and 98.47% inhibition against *S. aureus* and *E. coli*, respectively.

## 1. Introduction

Semiconductor photocatalytic materials are crucial in environmental remediation and sustainable energy applications due to their ability to harness solar energy to drive chemical reactions [1,2]. In recent decades, they have been employed in a variety of applications, including the treatment of organic pollutants [3,4], the production of hydrogen by photolysis of water [5,6], the reduction of CO_2_ [7,8], and the degradation of microbials [9,10]. These materials typically include metal oxides such as titanium dioxide (TiO_2_), zinc oxide (ZnO), and other metal sulfides and nitrides.

Titanium dioxide (TiO_2_) is one of the most commonly used and highly efficient photocatalysts, which is characterized by low cost, non-toxicity, good stability, and high photocatalytic activity [11,12,13]. When TiO_2_ is exposed to light with energy equal to or greater than its forbidden band energy (Eg), electrons in the valence band (VB) are excited to the conduction band (CB). At the same time, holes are left in the valence band, forming electron (e^−^) and hole (h^+^) pairs. When exposed to oxygen and water vapor, the TiO_2_ will produce hydroxyl radicals (·OH), hydrogen peroxide (H_2_O_2_), and reactive oxygen species (ROS), such as the superoxide radical (O_2_·^−^), which can rapidly decompose organic pollutants or bacteria [14]. However, TiO_2_ has a high forbidden band gap (Eg 3.2 eV), which renders it primarily sensitive to UV light and less applicable to simulated sunlight [15,16]. Its electronic and energy band structures can be modified to enhance its simulated sunlight responsiveness [17].

In recent years, numerous methods have been employed to modify TiO_2_ to enhance its photocatalytic performance. These include ion doping [18,19], noble metal deposition (Pt, Pd, Au, Ag, etc.) [20,21], and semiconductor compounding to form p-n heterojunctions [22,23]. Among these methods, ion doping modification is regarded as a competent technique that can be employed to achieve an efficient response to solar irradiation by modifying the electronic structure of TiO_2_ nanoparticles. Doping is a highly effective technique for suppressing the recombination of light-generated carriers and enhancing photocatalytic activity. By introducing dopant ions, TiO_2_ can extend its active wavelength range from UV to visible light, thereby improving solar photocatalytic efficiency [24,25]. Dopants create new energy levels, reduce the band gap energy, broaden solar absorption, and enhance TiO_2_ nanoparticles’ photocatalytic activity under simulated sunlight [26]. Additionally, dopant ions can act as shallow traps for electrons or holes, significantly reducing electron–hole recombination rates and prolonging carrier lifetimes, further boosting photocatalytic performance [27,28]. Transition metal ions, including Fe^3+^, Cr^3+^, Co^2+^, and Ni^2+^, have been employed to dope modified TiO_2_ nanoparticles, to enhance their photocatalytic performance [29,30,31,32]. Nevertheless, some studies have indicated that during the photocatalytic degradation of organic pollutants in water, the presence of metal cations in nano-TiO_2_ can result in the formation of composite pollutants with organic pollutants, which may subsequently contribute to environmental degradation [33].

Selenium is an essential component of numerous antioxidant enzymes in living organisms and exhibits several advantageous properties, including high bioactivity, biocompatibility, and environmental friendliness [34,35]. It has been demonstrated that selenium has good broad-spectrum photosensitizing properties and forms p-n heterojunctions with nano-TiO_2_ to extend the photo response range of nano-TiO_2_ [36,37]. While Se exists in various chemical valencies, including Se^4+^, Se^6+^, and Se^2−^, some calculations have shown that Se^4+^ doping of TiO_2_ can introduce additional electronic states in the band gap [38]. Gurkan et al. [38] prepared Se^4+^doped TiO_2_ photocatalysts with varying concentrations using an impregnation method to investigate the photocatalytic degradation kinetics. The results demonstrated that 0.50% Se^4+^doped TiO_2_ exhibited the highest degradation rate of 4-nitrophenol. Wei et al. [39] modified nano TiO_2_ with Se^4+^ and increased the doping concentration of selenium in nano TiO_2_ to 17.1%. The band gap width of the nano-TiO_2_ was significantly reduced, resulting in enhanced photocatalytic activity under solar irradiation.

Traditional photocatalysts are typically in powder form, posing challenges for separation and recovery from wastewater, leading to resource wastage and environmental pollution. Polyester (PET) fibers, valued for their high strength, corrosion resistance, heat tolerance, and affordability, serve as an ideal matrix for loading nano-TiO_2_ photocatalysts. This integration facilitates the recyclability of photocatalytic materials and reduces overall costs [40,41]. In this study, the synthesis of the Se^4+^@TiO_2_ photocatalytic material was achieved through the sol–gel method. PET fabrics were pretreated with plasma technology, which created active sites for the Se^4+^@TiO_2_ to enhance the bonding fastness to PET fabrics. The subsequent preparation of the Se^4+^@TiO_2_/PET material was conducted by an impregnation method. The surface morphology, chemical composition, crystal structure, and surface properties of the PET fabrics were analyzed. Meanwhile, the alterations in the surface morphology, chemical composition, and crystal structure of polyester fabrics following finishing were analyzed, thereby providing a theoretical foundation for the utilization of Se^4+^modified nano-TiO_2_ in the field of photocatalytic antimicrobial textiles.

## 2. Results and Discussion

### 2.1. SEM Analyses

**Figure 1:**
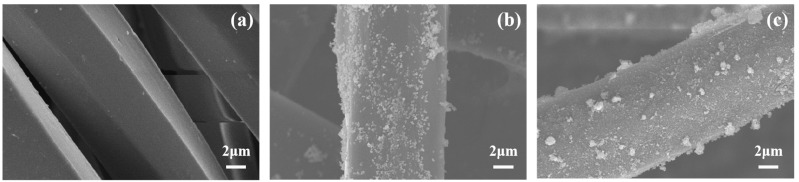
Scanning electron microscopy images of composite photocatalytic materials: (**a**) untreated PET; (**b**) TiO_2_/PET; (**c**) Se^4+^@TiO_2_/PET.

Figure 1 illustrates the surface morphology of the PET fabric before and after treatment. Figure 1a depicts an SEM image of the PET fabric with 5000 times magnification. This image reveals that the surface of the untreated PET fabric is smooth and free of impurities. Figure 1b depicts an SEM image of TiO_2_/PET with 5000 times magnification. It can be observed that the nano-TiO_2_ is more uniformly distributed on the surface of the PET, with an average size of approximately 100–120 nm. Figure 1c depicts an SEM image of Se^4+^@TiO_2_/PET with 5000 times magnification, which has been synthesized by the sol–gel method. The average size of the Se^4+^@TiO_2_ is 60–80 nm. There is local aggregation on the surface of the PET fabric due to the easier aggregation of nanoparticles.

### 2.2. EDS Analyses

**Figure 2:**
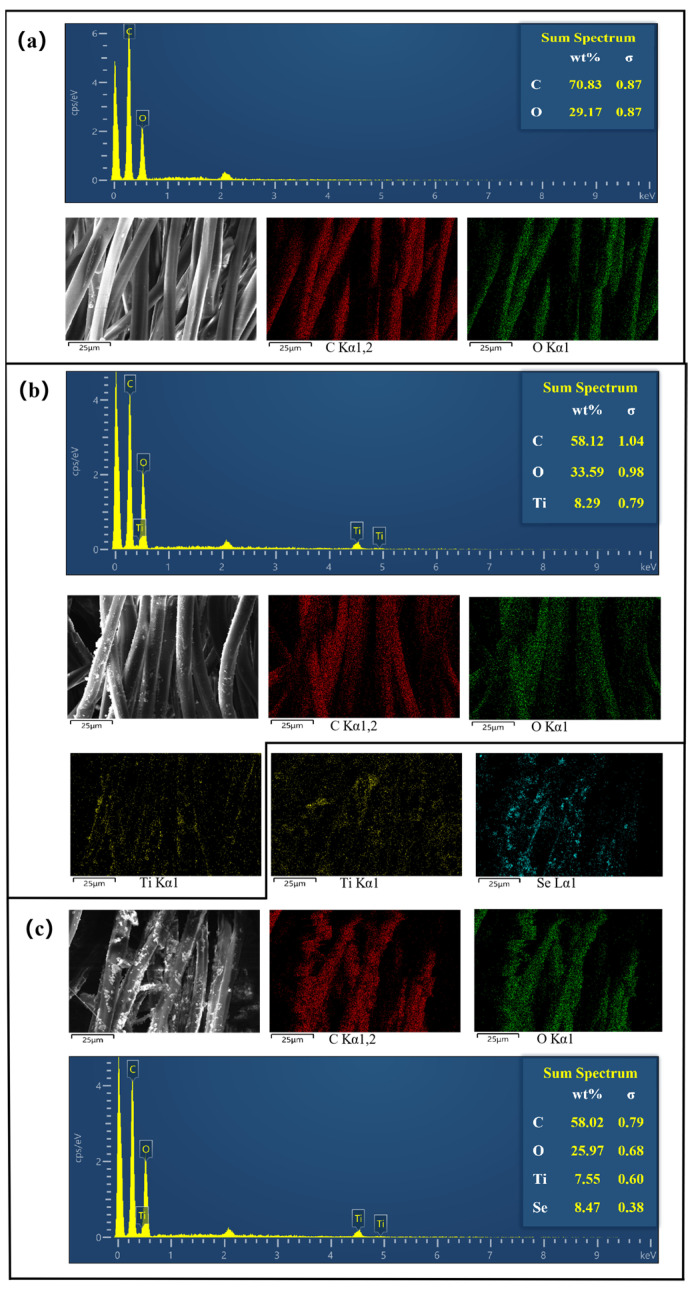
Dispersion of photocatalytic composite material elements: (**a**) untreated PET; (**b**) TiO_2_/PET; (**c**) Se^4+^@TiO_2_/PET.

Figure 2 illustrates the surface elemental distribution of the PET fabric before and after the treatment process. The elemental composition of the untreated PET fabric consists primarily of carbon and oxygen, with relative contents of 70.83% and 29.17%, respectively, as shown in Figure 2a. In Figure 2b, the elemental distribution of the TiO_2_/PET composite reveals an increased proportion of titanium (Ti), which results from the incorporation of TiO_2_ onto the PET surface, with a relative Ti content of 8.29%. Figure 2c demonstrates a uniform distribution of Ti and Se elements across the PET fabric surface. The results indicate that the mass percentages (wt%) of Ti and Se are 7.55% and 8.47%, respectively. The presence of these elements confirms the successful loading of TiO_2_ and Se.

### 2.3. XRD Analyses

**Figure 3:**
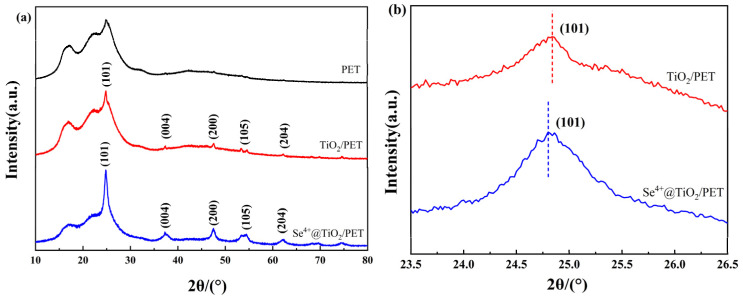
X-ray diffraction pattern images of composite photocatalytic materials: (**a**) XRD full spectrum; (**b**) Diffraction peak of (101) crystal plane.

The crystalline structures of the three materials, PET, TiO_2_/PET, and Se^4+^@TiO_2_/PET, are depicted in Figure 3. In Figure 3a, the diffraction peaks of the three curves, indicated by the circles at 17.2°, 22.6°, and 25.1°, correspond to the diffraction peaks of the PET [42]. The diffraction peaks of TiO_2_/PET, indicated by the five positions of 25.2°, 37.5°, 47.8°, 53.5°, and 62.3°, correspond to the crystallographic planes of (101), (004), (200), (105), and (204) in PDF#71–1168, which are the diffraction peaks of anatase TiO_2_ [43,44]. As illustrated in Figure 3b, the XRD diffractogram of Se^4+^@TiO_2_/PET also exhibits the characteristic diffraction peaks of anatase-phase TiO_2_, thereby indicating that Se^4+^ does not affect the physical phase of nano TiO_2_. Moreover, the diffraction peaks of Se^4+^@TiO_2_/PET at the (101) crystal plane exhibit a slight shift to a lower angle, which indicates that Se^4+^ enters the substitution sites of the TiO_2_ crystal structure [45,46]. The Deby–Scherrer formula Equation (1) was employed for the calculation of the crystal size [47]:

(1) $$D=\frac{k\lambda }{\beta \cos\theta }$$

where *D* is the crystal size, nm; *k* is the Scherrer constant and taken as 0.89; *λ* is the X-ray wavelength and taken as 0.15406 for Cu kα; *β* is the full width at the half maximum intensity of the peak (FWHM); *θ* is the angle of diffraction, 2*θ*/2. From this equation, the mean diameter of the TiO_2_ nanoparticles was determined to be 100 nm, while that of the Se^4+^@TiO_2_ particles was found to be 60 nm. Concurrently, calculations demonstrated that the size of the Se^4+^@TiO_2_ crystals exhibited a reduction in comparison to that of TiO_2_, accompanied by an increase in disorder or defects within the crystals. This phenomenon can be attributed to the lattice deformation caused by the doping of Se^4+^ ions, which results in the formation of new defects in the TiO_2_ grains, thereby reducing the orderliness of the material [38].

### 2.4. XPS Elemental Analysis

**Figure 4:**
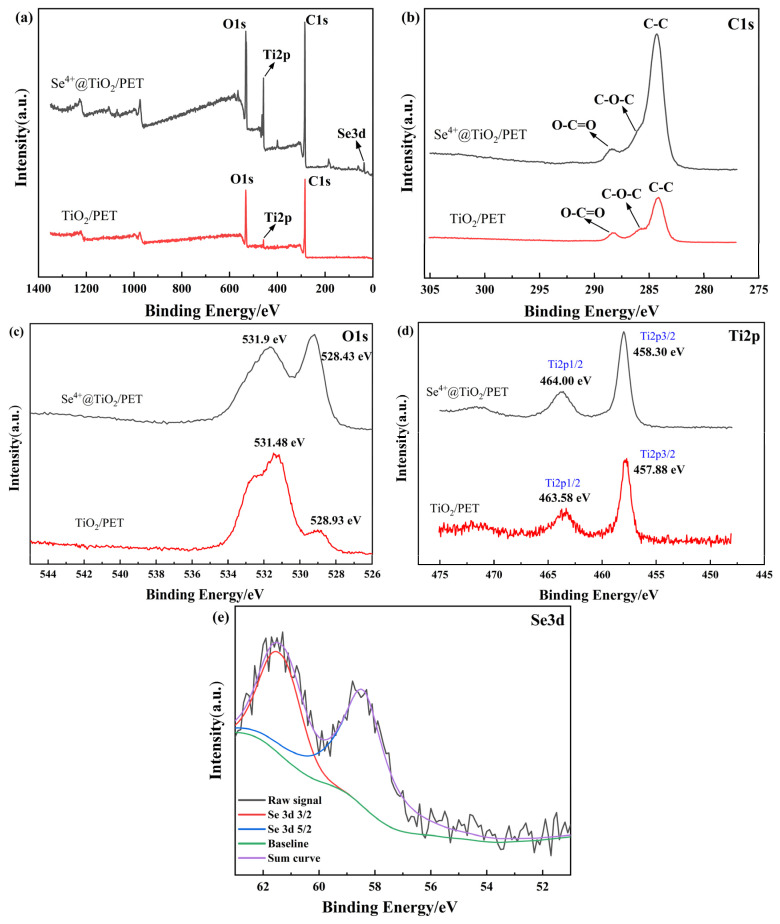
XPS image of TiO_2_/PET and Se^4+^@TiO_2_/PET: (**a**) wide spectrum; (**b**) C1s spectrum; (**c**) O1s spectrum; (**d**) Ti2p spectrum; (**e**) Se3d spectrum.

Figure 4a illustrates the full peaks of TiO_2_/PET, which contain C 1s, O 1s, and Ti 2p signals, while those of Se^4+^@TiO_2_/PET contain C 1s, O 1s, Ti 2p, and Se 3d signals. The peaks of the C and O elements are primarily derived from PET fabrics [48]. Figure 4b illustrates the binding energy of C 1s. The peaks observed in the two curves in the figure correspond to the O-C=O, C-O-C, and C-C bonds in the PET, respectively. Figure 4c depicts the photoelectron spectra of O 1s. Peaks at 531.48 eV in TiO_2_/PET may be attributed to surface hydroxyl (-OH) groups and another peak at 528.93 eV is caused by lattice oxygen. Figure 4d displays the photoelectron spectra of elemental titanium. The peaks observed at 457.88 eV and 463.58 eV for TiO_2_/PET correspond to the Ti 2p3/2 and Ti 2p1/2 signals, respectively. The difference in binding energies between the two peaks is 5.7 eV, which indicates the presence of Ti^4+^ in the material [49,50,51].

The Ti 2p3/2 and Ti 2p1/2 spectra in Se^4+^@TiO_2_/PET exhibit two major characteristic peaks at 458.30 eV and 464.00 eV, respectively. The difference in binding energy between the two peaks is 5.7 eV, which also suggests that the elemental valence state of Ti in Se^4+^@TiO_2_/PET is also +4 valence. Further analysis demonstrates that Se^4+^ doping into TiO_2_ results in a shift of the Ti 2p3/2 peak from 457.88 eV to 458.30 eV, while the Ti 2p1/2 peak shifts from 463.58 eV to 464.00 eV. Both peaks exhibit a shift towards higher binding energy. Any shift greater than 0.2 eV indicates a genuine change in the XPS spectrogram, suggesting the emergence of novel species in the XPS spectrogram of the TiO_2_ catalyst surface [52]. These shifts are due to the fact that Se^4+^ occupies the position of Ti^4+^ after Se^4+^ doping of TiO_2_. The electron transfer from Ti^4+^ to Se^4+^ decreases the electron cloud density around the Ti atom’s nucleus, increases its positive charge density, and raises the binding energy, indicating successful doping of Se^4+^ into the TiO_2_ lattice [38]. Figure 4e presents the Se 3d spectra of Se^4+^@TiO_2_/PET, and the two prominent Se 3d peaks are evident in the image. In Figure 4e, the red line represents Se 3d 3/2 while the blue line represents Se 3d 5/2, where the Se 3d 5/2 peak corresponds to Se^4+^.

### 2.5. UV–Vis and PL Analyses

**Figure 5:**
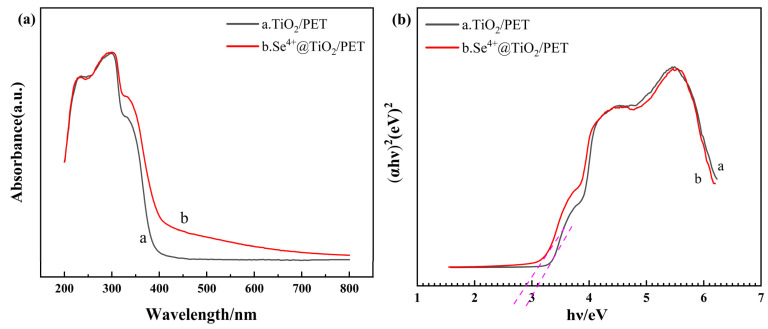
(**a**) UV–vis absorption spectra of TiO_2_/PET and Se^4+^@TiO_2_/PET; (**b**) The relationship curve between (*αhv*)^2^ and *hv* of TiO_2_/PET and Se^4+^@TiO_2_/PET.

In order to ascertain the optical properties of the photocatalytic composites, UV–visible and PL tests were conducted [53]. The energy band structure of the semiconductor materials can be obtained analytically through UV–Vis absorption spectroscopy. Figure 5a illustrates the UV–Vis absorption spectra of TiO_2_/PET and Se^4+^@TiO_2_/PET, spanning a wavelength range of 200–800 nm. Compared to TiO_2_/PET, Se^4+^@TiO_2_/PET exhibited enhanced absorption properties within the visible light range. The absorption spectra of Se^4+^@TiO_2_/PET exhibited a redshift within the simulated sunlight range, suggesting an increase in its photocatalytic activity within the simulated sunlight range. This was attributed to the change in the conduction and valence bands in Se^4+^@TiO_2_/PET [54]. The photocatalytic effect can be formulated as follows (Equation (2)) [55]:

(2) $${\left(\alpha h\nu \right)}^{2}=A(h\nu -Eg)$$

where *A* is a constant, *α* is the light absorption coefficient, *h* is Planck’s constant, *ν* is the optical frequency, and *Eg* is the forbidden band gap of the material.

Figure 5b plots (*αhν*)^2^ versus *hν* and extrapolates the linear portion of the plot to the energy axis to determine the energy band gap. The forbidden band gap of TiO_2_/PET is 3.1 eV, while the relative forbidden band gap of Se^4+^@TiO_2_/PET is 2.9 eV. The narrowing of the band gap of Se^4+^@TiO_2_/PET reduces the rate of photogenerated electron and hole complexation. This, in turn, leads to a shift in the absorption threshold (400 nm for TiO_2_/PET and 476 nm for Se^4+^@TiO_2_/PET) towards the visible light region, thereby broadening the range of light utilization. Consequently, the simulated solar irradiation photocatalytic activity of the composite photocatalytic materials is enhanced by the incorporation of Se^4+^ into TiO_2_.

**Figure 6:**
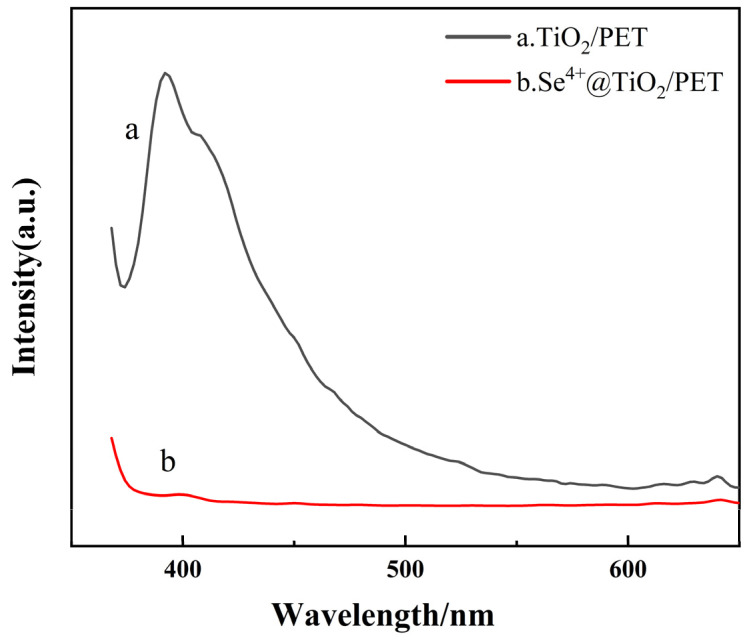
Photoluminescence spectra of TiO_2_/PET and Se^4+^@TiO_2_/PET.

The photogenerated electron–hole complexation and trapping processes of each catalyst were analyzed by photoluminescence spectroscopy, which enables the characterization of the information about the separation and complexation of photogenerated carriers in semiconductors [56,57]. A reduction in spectral intensity indicates a diminished photogenerated electron–hole recombination reaction, which is associated with enhanced photocatalytic performance. Figure 6 illustrates the photoluminescence spectra of TiO_2_/PET and Se^4+^@TiO_2_/PET. Figure 6 reveals a notable decline in the photoluminescence intensity of Se^4+^@TiO_2_/PET compared to TiO_2_/PET. The electron–hole complexation rate is proportional to the photoluminescence spectral intensity, and the faster the complexation rate, the stronger the spectral intensity. Conversely, a lower photoluminescence (PL) intensity indicates that the photogenerated electrons are trapped and safely transferred to the surface of the photocatalytic material, where they react with adsorbed oxygen or water molecules [58,59]. The lower PL intensity of Se^4+^@TiO_2_/PET suggests a lower rate of complexation, which in turn implies better photocatalytic activity.

### 2.6. Photocatalytic Performance Analysis

**Figure 7:**
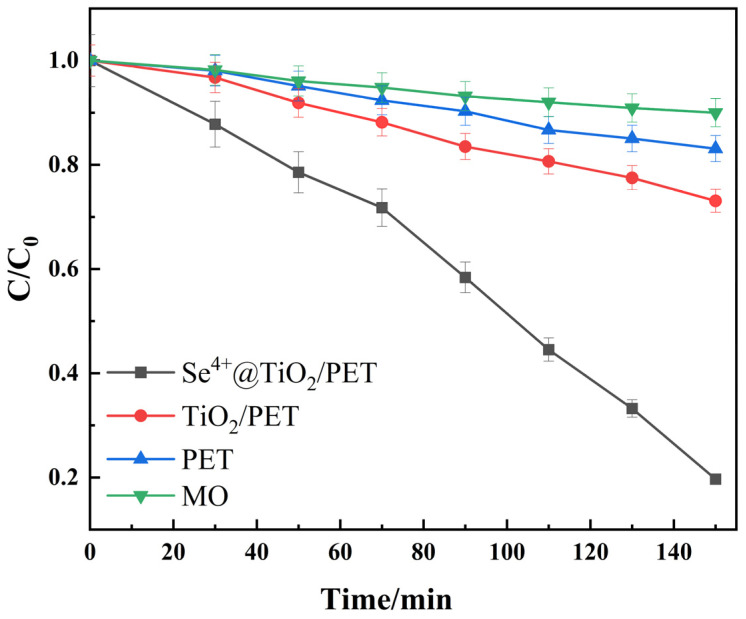
Degradation curves of methyl orange by TiO_2_/PET and Se^4+^@TiO_2_/PET under simulated sunlight irradiation.

The photocatalytic activities of TiO_2_/PET and Se^4+^@TiO_2_/PET on a methyl orange (MO) solution were compared under simulated sunlight irradiation, as illustrated in Figure 7. The samples were magnetically stirred in a dark environment for 30 min to achieve adsorption equilibrium between the photocatalysts and the MO solution. Following a 30 min stirring period in the absence of light, the MO with the composite photocatalyst was subjected to irradiation with an xenon lamp for a duration of 120 min, and the supernatant was taken every 20 min to test the absorbance. The control sample was a 20 mg/L MO, and the degradation efficiency was calculated. Figure 7 illustrates that in the absence of any photocatalytic material, the MO undergoes slight self-degradation, with a degradation efficiency of 10%. The addition of PET results in an increase in the removal efficiency of MO by PET, reaching 17%. Following the loading of nano-TiO_2_, the removal efficiency of MO by TiO_2_/PET was found to be 19%, while that of MO by Se^4+^@TiO_2_/PET was 81%. In comparison to TiO_2_/PET, the photocatalytic performance of Se^4+^@TiO_2_/PET for MO was found to be significantly enhanced.

The photocatalytic degradation of MO was analyzed according to the Langmuir–Hinshelwood kinetic model [60,61] with the kinetic Equation (3):

(3) $$r=-\frac{dC}{dt}=\frac{k_{r}k_{s}C_{0}}{1+k_{s}C_{0}}$$

where *C*_0_ is the initial concentration of the dye, *k_r_* is the reaction rate constant, *k_s_* is the apparent adsorption constant, and *t* is the reaction time. When the concentration is low, the *k_s_C*_0_ value is deemed insignificant, allowing the reaction rate to be expressed through a pseudo first-order model (Equation (4)):

(4) $$-\frac{dC}{dt}=k_{r}k_{s}C_{0}=K_{app}C_{0}$$

**Figure 8:**
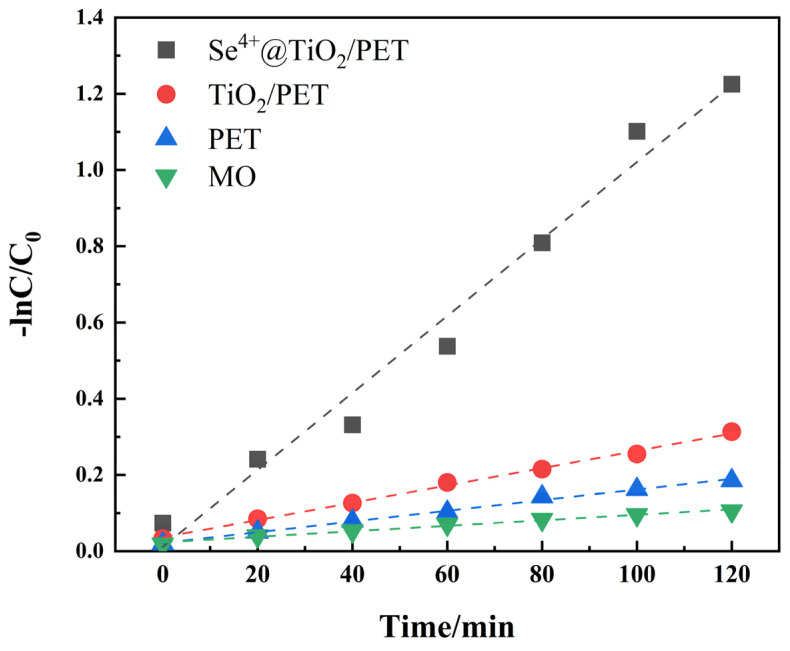
Degradation kinetics curve of methyl orange.

The integration of Equation (3) yields the following result (Equation (5)):

(5) $$-ln(\frac{C}{C_{0}})=K_{app}t$$

where *K_app_* is defined as the slope of the linear regression equation. The pseudo-primary kinetic equation is used to linearly fit the experimental data, resulting in the apparent rate constants *K_app_* and the correlation coefficients *R*^2^ for different samples, as shown in Table 1. In conjunction with Figure 7 and Figure 8, it can be observed that following 120 min of simulated sunlight irradiation, Se^4+^@TiO_2_/PET exhibited the most pronounced degradation efficiency of MO, reaching 81%. This was accompanied by an apparent rate constant of 0.0101 min^−1^, which was 4.4 times higher than that of TiO_2_/PET. The results indicated that the photocatalytic degradation followed a pseudo-primary kinetic equation (Figure 8).

**Figure 9:**
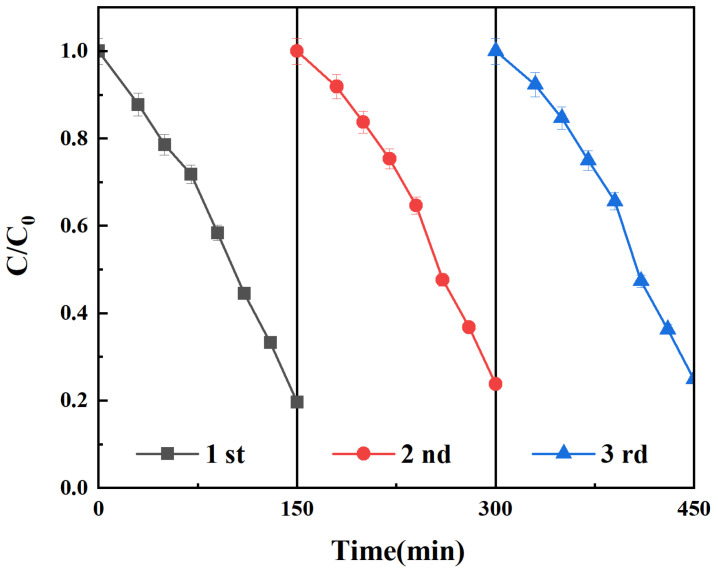
The reusability of Se^4+^@TiO_2_/PET by degradation of methyl orange.

To evaluate the reusability of the Se^4+^@TiO_2_/PET composites, the photocatalytic degradation tests were performed on the samples for three times after recovery, collection, washing, and drying as illustrated in Figure 9. The results showed that the degradation efficiency changed from 81% to 76% by Equation (13), indicating the good recyclability and stability of the composites.

### 2.7. EIS Analyses

**Figure 10:**
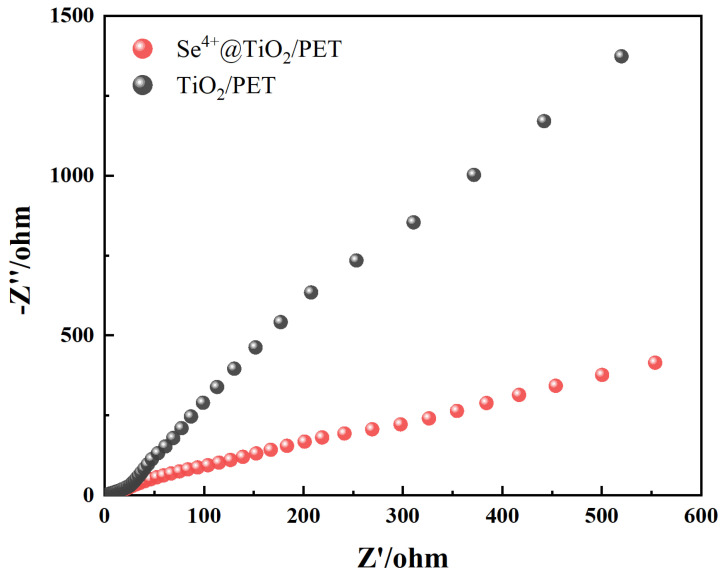
Electrochemical impedance spectra of TiO_2_/PET and Se^4+^@TiO_2_/PET.

As demonstrated in Figure 10, the electrochemical impedance spectra of TiO_2_/PET and Se^4+^@TiO_2_/PET reflect the interfacial resistance during the electron-leaping process in these samples. In comparison with TiO_2_/PET, the composite photocatalytic material Se^4+^@TiO_2_/PET exhibited a significantly reduced circular radius, indicating higher electronic conductivity. This is attributed to the lower resistance of the photocharge transfer at the interface between the Se^4+^@TiO_2_ photocatalyst and the electrolyte [62,63].

### 2.8. Radical Scavenger Analysis

Table 2 demonstrates the degradation efficiency of Se^4+^@TiO_2_/PET on a methyl orange solution with the addition of various free radical scavengers. In the absence of free radical scavengers, the degradation efficiency of methyl orange was found to be 81%. The addition of 10 mmol/L isopropanol (IPA) resulted in the capture of the hydroxyl radical (·OH), which led to a degradation rate of 77.28% for methylene orange. The addition of 1 mmol/L p-benzoquinone (BQ) resulted in the capture of the superoxide radical (O_2_·^−^), with a subsequent reduction in the degradation rate of methyl orange to 47.11%. The degradation rate of methyl orange was 72.64% after the addition of 10 mmol/L EDTA-2Na, which served to capture the hole (h^+^). It can thus be inferred from the capture experiments that the primary substance responsible for dye degradation and bacterial inactivation is the superoxide radical (O_2_·^−^).

### 2.9. Antimicrobial Performance Analysis

**Figure 11:**
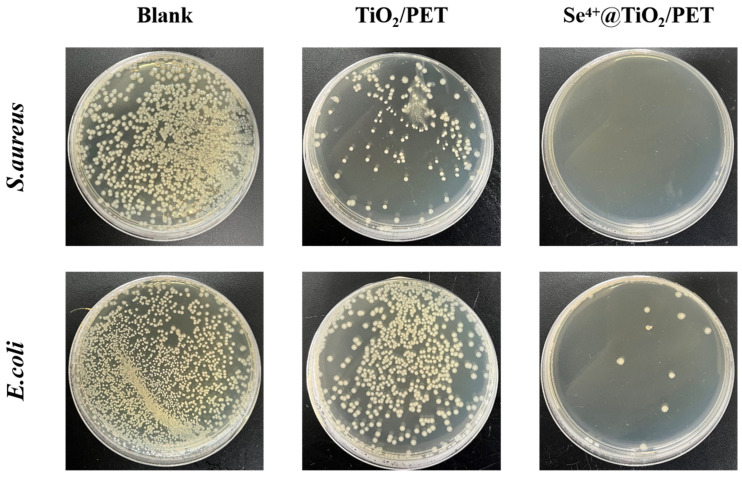
Antibacterial activity of TiO_2_/PET and Se^4+^@TiO_2_/PET against *E. coli* and *S. aureus*.

The antibacterial property test of TiO_2_/PET and Se^4+^@TiO_2_/PET against *Escherichia coli* (*E. coli*) and *Staphylococcus aureus* (*S. aureus*) are presented in Figure 11 and Table 3, respectively. As illustrated in Table 3, the inhibition rates of TiO_2_/PET against *E. coli* and *S. aureus* were relatively low, at 58.04% and 88.31%, respectively. Additionally, TiO_2_ exhibited a toxic effect on bacteria under dark conditions, which was postulated to be potentially due to the equilibrium phase prior to the initiation of photocatalysis. These findings are consistent with previous studies that have demonstrated the inhibition rates of Se^4+^@TiO_2_/PET against *S. aureus* and *E. coli* were 99.99% and 98.47%, respectively, with significant antibacterial effects [64,65]. This indicates that the Se^4+^@TiO_2_/PET material exerts its antimicrobial activity by stimulating the highly active ROS generated by the Se^4+^@TiO_2_ composite catalyst, resulting in irreversible damage to microbial cell walls and membranes, leading to leakage of microbial cell contents, impaired cellular functions, and ultimately inactivation and death of the microorganisms [66,67,68].

### 2.10. The Photocatalytic Antibacterial Reaction Mechanism Analysis

**Figure 12:**
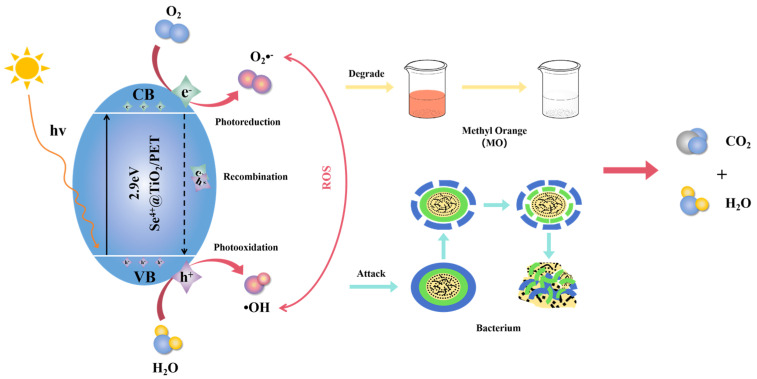
Se^4+^@TiO_2_/PET UV–Vis photocatalytic degradation and antimicrobial mechanism map.

Figure 12 presents a diagram of the antimicrobial mechanism of UV–visible photocatalytic degradation of Se^4+^@TiO_2_/PET. This diagram can explain the principle of degradation of MO and inhibition of bacteria by the material. When Se^4+^@TiO_2_ is exposed to light with an adsorption energy greater than or equal to its band gap (Eg), electrons (${e_{CB}}^{-}$) filling the valence band (VB) are excited and jump into the empty conduction band (CB), forming holes (${h_{VB}}^{+}$) in the VB (Equation (6)). These excited electrons and holes are usually called carriers. Most of the electron–hole pairs produced by absorbing light recombine to produce emission energy (Equation (7)). In contrast, when the light-generated carriers do not recombine and the electron–hole pairs separate, the electrons and holes are transferred to the catalyst surface, where they are captured by the surface-active sites and drive the redox reaction. The entire photocatalytic reaction can be decomposed into two half-reactions: electron-induced reduction and hole-induced oxidation. Photogenerated electrons can react with dissolved oxygen molecules (O_2_) in aqueous solution to form superoxide radical anions (O_2_·^−^) (Equation (8)), while holes can interact with H_2_O molecules on the surface of catalyst particles to form hydroxyl radicals (·OH) (Equation (9)). All of these generators are collectively referred to as highly reactive oxidants (ROS), which can be further involved in the oxidative degradation of organic pollutants by mineralizing them into CO_2_ and H_2_O (Equations (10) and (11)) [69,70]. In addition, it is also important to consider that the presence of ROS may lead to the damage of the molecular as well as organic polymer matrix.

The energy level of Se^4+^ (2.27 eV) is a determining factor in the high photocatalytic activity of the composite. When the energy level of the Se^4+^doped ion lies below the conduction band edge, it traps the excited electrons; when the energy level is above the valence band edge, the electrons can burst the photogenerated holes, the center of which can act as an electron or hole trap, thus temporarily separating the light-generated carriers [34]. Furthermore, the photocatalytic reaction is proportional to the number of photons absorbed by the photocatalyst. The doping of titanium dioxide with Se^4+^ has been observed to enhance the light-absorbing properties of the material. This phenomenon is associated with an increase in the generation of electron and hole pairs on the surface of Se^4+^@TiO_2_/PET, which in turn facilitates the participation of these charge carriers in redox reactions. This process has been demonstrated to enhance the degradation efficiency of methyl orange [71].

(6) $${Se}^{4+}@{TiO}_{2}+hv\to {e_{CB}}^{-}+{h_{VB}}^{+}$$

(7) $${e_{CB}}^{-}+{h_{VB}}^{+}\to energy$$

(8) $$O_{2}+{e_{CB}}^{-}\to {O_{2}\cdot }^{-}$$

(9) $$H_{2}O+{h_{VB}}^{+}\to \cdot OH+H^{+}$$

(10) $${O_{2}\cdot }^{-}+MO\to {CO}_{2}+H_{2}O$$

(11) $$\cdot OH+MO\to {CO}_{2}+H_{2}O$$

Several reports have confirmed that bacteria can be decomposed and mineralized by TiO_2_ during the photocatalytic process [72,73]. TiO_2_ has the capacity to degrade the impurities into less toxic terminal compounds or even mineralize all of them into CO_2_ and H_2_O. While selenium itself has high antimicrobial properties [74], the incorporation of Se^4+^ into TiO_2_ will enhance the antibacterial performance of the composite photocatalytic materials. The bacterial inhibition mechanisms of photocatalytic antimicrobial composites can be broadly classified into three categories [75]: (a) biological processes, which lead to the internalization of the nanoparticles in *bacteria* through ion channels or proteins in the cell wall; (b) physical processes, which include the adsorption of photocatalytic antimicrobial composites on the cell surface; and (c) chemical phenomena, which are either generated by ROS or due to the removal from the toxic effects of ionic substances leached from the nanoparticles. Upon direct contact between Se^4+^@TiO_2_/PET and the bacterial surface, the peptidoglycan of the bacterial cell wall was destroyed by the ROS generated in the photocatalytic reaction. Additionally, the cell membrane components were peroxidized during the photocatalytic process, which led to an increase in the permeability of the cell membrane, destroying its integrity. Ultimately, this resulted in the inactivation of the bacteria and further decomposition of the bacterial residues (Equation (12)) [76].

(12) $$\cdot OH+Bacterium\to {CO}_{2}+H_{2}O$$

## 3. Materials and Methods

### 3.1. Materials

The polyester knitted fabric was supplied by Jiangsu Kuangda Technology Group Co., Ltd. (Changzhou, China) with a grammage weight of 230 g/m^2^. The reagents used in this study were titanium dioxide (TiO_2_, Aladdin Biochemical Technology Co., Ltd., Shanghai, China), glacial acetic acid (Hubao Chemical Reagent Co., Ltd., Yangzhou, China), tetrabutyl titanate (Yuanye Biotechnology Co., Ltd., Shanghai, China), selenium dioxide (SeO_2_, McLean Biochemical Ltd., Shanghai, China), anhydrous ethanol (Runjie Chemical Reagent Co., Ltd., Shanghai, China), acetone (Lingfeng Chemical Reagent Co., Ltd., Shanghai, China), and methyl orange (Yuanye Biotechnology Co., Ltd., Shanghai, China), all of which were of analytical grade. Deionized water was used for all experiments.

### 3.2. Preparation of Se^4+^@TiO_2_ Composite Photocatalysts

First, 20 mL of anhydrous ethanol was mixed with 5 mL of glacial acetic acid under magnetic stirring at 600 r/min, and 10 mL of tetrabutyl titanate was added drop by drop and recorded as solution A. The solution was mixed with 5 mL of deionized water and 0.33 g of SeO_2_ under magnetic stirring until SeO_2_ was completely dissolved. Then 20 mL of anhydrous ethanol was mixed with 0.5 mL of deionized water and 0.33 g of SeO_2_ with magnetic stirring until the SeO_2_ was completely dissolved, recorded as solution B. Solution B was mixed with 0.5 mL of deionized water and 0.33 g of SeO_2_ with magnetic stirring until the SeO_2_ was completely dissolved. Solution B was added into solution A under magnetic stirring at 1500 r/min, the resulting sol was aged for 12 h to obtain a wet gel, and the wet gel was dried at 80 °C for 24 h to obtain a dry gel. Subsequently, the dry gel was pulverized and calcined in a resistance furnace at 500 °C for a period of 2 h, resulting in the production of a photocatalyst doped with a 10% molar ratio of Se^4+^ to TiO_2_.

### 3.3. Preparation of Se^4+^@TiO_2_-Loaded PET Fabrics

The PET fabrics were dispersed in a 1:1:1 mixture of ethanol, acetone, and deionized water at room temperature for 30 min using a 70 W model KH-250DE ultrasonic disperser produced by Kunshan Hexiang Ultrasonic Instrument Co. Ltd. (Kunshan, China), and then placed into the dispersed solution and shaken in a water bath at 60 °C for 2 h. The PET fabrics were taken out and rinsed with deionized water more than 5 times, and dried at 60 °C. The pretreated PET fabrics were cut into 10 cm × 10 cm and placed into a model PDC-VCG-2 plasma chamber manufactured by Harrick Scientific Products, Inc. (New York, NY, USA) for 2 min; the plasma power supply voltage was 220 V, frequency was 50 Hz, and the processing power was 18 W. Then 0.006 mol of Se^4+^@TiO_2_ or TiO_2_ photocatalyst was dispersed in 50 mL of deionized water by ultrasonication for 30 min, respectively, followed by the addition of 1 g of plasma-treated PET fabrics to ensure the identical loading of photocatalysts deposited on the fibers. After that, the suspension was oscillated in a water bath for 2 h. The PET fabrics were then removed and rinsed more than 5 times with deionized water and dried at 60 °C.

### 3.4. Characterization

Various analytical techniques were carried out on the experimental samples in order to investigate their surface morphology, crystal structure, chemical composition, optical characteristics, band gap width, photocatalytic activity, and antimicrobial properties. Specifically, the surface morphology of the materials was analyzed using a ZEISS Gemini SEM 300, Oberkochen, Germany, field emission scanning electron microscope (SEM) with an accelerating voltage of 5 kV. The elemental composition of the material surface was analyzed using a Genesis XM series X-ray energy dispersive spectrometer (EDS) on a Carl Zeiss EVO15 instrument from Oberkochen, Germany. The crystalline phase structure of the materials was analyzed using an X-ray diffractometer (XRD) model H-12, manufactured by Rigaku Corporation, Tokyo, Japan, operated at a voltage of 40 kV and a current of 100 mA with a 2θ scanning range of 10° to 80°. An X-ray photoelectron spectrometer (XPS) model K-Alpha from Thermo Fisher Scientific Inc., Waltham, MA, USA was used to analyze the elemental species, chemical composition, and information about the electronic structure of the material surfaces, and the elements tested were C, Ti, O, and Se. A UV–visible diffuse reflectance spectrophotometer (UV–Vis) model UH4150 from Hitachi Ltd., Tokyo, Japan was used to analyze the UV–Vis absorption spectra of the samples with the wavelength of 200–800 nm at 1 nm intervals at a scanning speed of 1200 nm/min. The fluorescence spectra of the samples were measured using a full-featured steady state/transient fluorescence spectrometer (PL) model FLS980 from Edinburgh Instruments Ltd., Livingston, UK, under 450 W xenon lamp irradiation. The electrochemical impedance spectroscopy (EIS) test was conducted using a CHI760 electrochemical workstation manufactured by Shanghai Chenhua Instrument Co. Ltd., Shanghai, China. The test was carried out in a standard three-electrode system in the frequency range of 0.1–10^5^ Hz with an amplitude of 10 mV, and the electrolyte was a 1 mol/L KOH solution.

### 3.5. Radical Trapping Analysis

The presence of the hydroxyl radical (·OH), superoxide anion (O_2_·^−^), and hole (h^+^) reactive species during the photocatalytic reaction was confirmed through the addition of 10 mM of IPA (a scavenger of ·OH), 1 mM of BQ (a scavenger of O_2_·^−^), and 10 mM of EDTA-2Na (a scavenger of h^+^). Subsequently, the degradation efficiency of Se^4+^@TiO_2_/PET on methyl orange was employed to ascertain the species responsible for dye degradation and bacterial inactivation.

### 3.6. Photocatalytic Degradation Performance Reaction Test

The methyl orange (MO) at a concentration of 20 mg/L was prepared as a model for organic pollutants. The composite photocatalytic materials were cut into pieces, submerged in 100 mL of the prepared MO, and stirred magnetically for 30 min in the dark to reach the adsorption equilibrium between the materials and MO. A PLS-SXE300+ xenon lamp with a power of 300 W and a wavelength of 320–780 nm manufactured by Beijing Porphyry Technology Co. Ltd. (Beijing, China) was used to simulate the sunlight source. The absorbance of the supernatant was measured at 20 min intervals and recorded. According to the Beer–Lambert law, there is a linear relationship between dye concentration and absorbed light, and the degradation efficiency of the photocatalytic material was calculated according to Equation (13) to analyze its simulated solar irradiation photocatalytic performance [77]:

(13) $$\eta =\frac{C_{0}-C_{t}}{C_{0}}\times 100\%=\frac{A_{0}-A_{t}}{A_{0}}\times 100\%$$

where η is the degradation efficiency of the material, %; *C*_0_ is the concentration of MO at the starting instant, mg/L; *C_t_* is the concentration of MO at the instant *t*, mg/L; *A*_0_ is the absorbance of MO at the starting instant; *A_t_* is the absorbance of MO at the instant t.

To evaluate the reusability of the Se^4+^@TiO_2_/PET composite, the Se^4+^@TiO_2_/PET was recycled, collected, washed, and dried. Subsequently, it was carried out three times under visible light irradiation.

### 3.7. Evaluation of Antimicrobial Performance

The antimicrobial properties of the materials were tested according to GB/T 20944.3–2008 [78] using the oscillation method. The test strains were gram-positive bacteria *S.aureus* and gram-negative bacteria *E.coli* [79]. The procedure was as follows: 0.75 g of a 0.5 cm × 0.5 cm sample was placed in a conical flask containing 70 mL of 0.03 mol/L phosphate buffered saline and 5 mL of diluted bacterial solution and incubated on a shaker at 24 °C for 24 h to form a culture solution. Then 1 mL of the diluted culture solution was evenly distributed in Petri dishes containing agar and then incubated in an inverted incubator at 37 °C for 24 h. At the end of the incubation period, the colonies produced were counted and the inhibition rate was calculated according to Equation (14) [80]:

(14) $$Y=\frac{N_{b}-N_{t}}{N_{b}}\times 100\%$$

where *Y* is the inhibition rate of the sample, %; *N_b_* is the number of colonies of the standard blank sample; *N_t_* is the number of colonies of other antimicrobial samples.

## 4. Conclusions

The Se^4+^@TiO_2_ photocatalytic material was prepared by the sol–gel method, and the plasma technique was employed to introduce free radicals on the surface of polyester fabric. This was performed to provide active sites for Se^4+^@TiO_2_ and to enhance its binding fastness to polyester fabric, thus preparing the Se^4+^@TiO_2_/PET composite photocatalytic material with an enhanced photocatalytic efficiency. The XRD results indicated that Se^4+^ had entered the substitution site of the TiO_2_ crystal structure in the composite photocatalytic material. The XPS results revealed that the valence state of Se was +4, and the shift of Ti 2p binding energy also indicated that Se^4+^ was doped into the TiO_2_ lattice. The PL intensity of the Se^4+^@TiO_2_/PET composite photocatalytic material was lower than that of TiO_2_/PET, suggesting a higher photocatalytic activity. In the UV–Vis spectrum, the forbidden band gap of TiO_2_/PET was 3.1 eV, while that of the Se^4+^@TiO_2_/PET composite was 2.9 eV. The bandgap of Se^4+^@TiO_2_/PET narrowed, the absorption threshold shifted to the visible light region, and the range of light utilization broadened. These observations indicate that Se^4+^ doping of TiO_2_ enhanced the simulated sunlight photocatalytic activity of composite photocatalytic materials. The photocatalytic and antimicrobial results demonstrated that Se^4+^@TiO_2_/PET exhibited a superior degradation efficiency of 81% for methyl orange solution in comparison to TiO_2_/PET. The antibacterial properties of Se^4+^@TiO_2_/PET were subsequently investigated, achieving 99.99% and 98.47% inhibition against *S. aureus* and *E. coli*, respectively. Nevertheless, the ROS generated by the photocatalyst have the potential to cause damage to the organic polymer carrier and degrade the matrix.

## Figures and Tables

**Table 1:** The constant of pseudo first-order kinetic equation for degradation of methyl orange under different photocatalysts.

Samples	*K_app_*	*R* ^2^
MO	0.0007	0.98443
PET	0.0014	0.99331
TiO_2_/PET	0.00227	0.99609
Se^4+^@TiO_2_/PET	0.0101	0.97474

**Table 2:** Degradation efficiency of Se^4+^@TiO_2_/PET for methyl orange under different radical scavengers.

Radical Scavenger	Blank	IPA	BQ	EDTA-2Na
Degradation efficiency %	81	77.28	47.11	72.64

**Table 3:** Antibacterial rates of TiO_2_/PET and Se^4+^@TiO_2_/PET against *E. coli* and *S. aureus*.

Samples	Concentration of Live *E. coli* Bacteria/(CFU·mL^−1^)	Inhibition Rate (%)	Concentration of Live *S. aureus* Bacteria/ (CFU·mL^−1^)	Inhibition Rate (%)
Blank	9.2 × 10^5^	n/a	8.9 × 10^5^	n/a
TiO_2_/PET	3.86 × 10^5^	58.04	1.04 × 10^5^	88.31
Se^4+^@TiO_2_/PET	1.4 × 10^4^	98.47	0	99.99

n/a: Not applicable.

## Data Availability

Data are contained within the article.
